# Solidification/Stabilization of Arsenic-Containing Tailings by Steel Slag-Based Binders with High Efficiency and Low Carbon Footprint

**DOI:** 10.3390/ma14195864

**Published:** 2021-10-07

**Authors:** Wei Gao, Zifu Li, Siqi Zhang, Yuying Zhang, Guoxiang Teng, Xiaoqi Li, Wen Ni

**Affiliations:** 1 School of Energy and Environmental Engineering, University of Science and Technology Beijing, Beijing 100083, China; 15201455635@163.com (W.G.); zifulee@aliyun.com (Z.L.); 2 Key Laboratory of High-Efficient Mining and Safety of Metal Mines, Ministry of Education, Beijing 100083, China; yuying.zhang@polyu.edu.hk (Y.Z.); 15110269699@163.com (G.T.); 3 Key Laboratory of Resource-Oriented Treatment of Industrial Pollutants, Beijing 100083, China; 4 School of Civil and Resource Engineering, University of Science and Technology Beijing, Beijing 100083, China; 5 Technology Center of HBIS Group Hansteel Company, Handan 056015, China; lixiaoqi@hbisco.com

**Keywords:** nonferrous metal tailings, steel slag-based binder, solidification/stabilization, arsenic, leaching risk

## Abstract

The disposal of nonferrous metal tailings poses a global economic and environmental problem. After employing a clinker-free steel slag-based binder (SSB) for the solidification/stabilization (S/S) of arsenic-containing tailings (AT), the effectiveness, leaching risk, and leaching mechanism of the SSB S/S treated AT (SST) were investigated via the Chinese leaching tests HJ/T299-2007 and HJ557-2010 and the leaching tests series of the multi-process Leaching Environmental Assessment Framework (LEAF). The test results were compared with those of ordinary Portland cement S/S treated AT (PST) and showed that the arsenic (As) curing rates for SST and PST samples were in the range of 96.80–98.89% and 99.52–99.2%, respectively, whereby the leached-As concentration was strongly dependent on the pH of the leachate. The LEAF test results showed that the liquid–solid partitioning limit of As leaching from AT, SST, and PST was controlled by solubility, and the highest concentrations of leached As were 7.56, 0.34, and 0.33 mg/L, respectively. The As leaching mechanism of monolithic SST was controlled by diffusion, and the mean observed diffusion coefficient of 9.35 × 10^−^^15^ cm^2^/s was higher than that of PST (1.55 × 10^−16^ cm^2^/s). The findings of this study could facilitate the utilization of SSB in S/S processes, replacing cement to reduce CO_2_ emissions.

## 1. Introduction

Nonferrous metals are an important basic material for the development of the Chinese economy [1]. However, the mining, processing, and smelting industry of non-ferrous metals is the most significant source of heavy metal and metalloid emissions in China (e.g., Hg, 53.6%; Cd, 88.9%; Pb, 81%; As, 61.8%) [2]. Mine wastewater, smelting slag, dust, and tailings are mainly piled up in tailings ponds, which poses high potential environmental risks [3]. It is imperative to develop effective and economically viable technologies to reduce pollution from the non-ferrous metal industry.

Backfilling is an ecological solidification/stabilization (S/S) method for the safe disposal of non-ferrous metal tailings, which could support underground goaf to prevent surface collapse, improve the recovery rate of resources, and prevent the release of contaminants from the tailings [4]. Ordinary Portland cement (OPC) is a traditionally used binder in backfilling S/S processes, but in the production of clinker, the raw materials need to be calcined in a rotary kiln at temperatures up to 1500 °C, leading to abundant emissions of CO_2_ in the process of decomposing the calcium carbonate into calcium oxide (CaO) and CO_2_. Cement accounts for 8% of total anthropogenic CO_2_ emissions [5]. The atmospheric CO_2_ concentration of about 420 ppm has already reached the halfway mark to 560 ppm which is double the preindustrial levels of 280 ppm [6]. Thus, any efforts to reduce carbon emissions are urgently needed. Research into developing low-carbon clinker-free binders or cement admixtures for S/S include ground granulated blast furnace slag (GGBFS) [7] as admixture, magnesium oxysulfate cement [8], red mud-enhanced magnesium phosphate cement [9], zero-valent iron or magnetic biochar [10], alkali-activated clay binder [11], fly ash-based Si-modified magnesia-style cement [12], fly ashes, and geopolymer binders surface-tailored with metakaolin composites [13]. However, few reports have been published on the application of steel slag [14,15,16,17,18]. Steel slag is a by-product of the steel-making process, known as overburnt clinker due to its mineral phase composition similar to cement clinker, of which 149.45 million tons were produced in China in 2019 with a comprehensive utilization rate of less than 30% [19]. The large accumulation of steel slag led to great pressure on the environment [20]. GGBFS is a by-product of the steel-making industry and is used as supplementary cementitious material. In the present study, steel slag-based binders (steel slag mass fraction ≥ 60 wt.%) (SSBs) composed of varying amounts of steel slag powder (SSP), GGBFS, and flue gas desulphurization gypsum (FGDG) were investigated as S/S binders. In the hydration process of SSBs, the relatively high alkalinity of SSP facilitates the breakage of Si–O and Al–O bonds in the GGBFS vitreous structure to form the anions ${SiO}_{4}^{4-}$ and ${AlO}_{4}^{5-}$ [21,22], which react with dissolved Ca^2+^ to form calcium silicoaluminate hydrate (C–A–S–H) gels [11,23]. The ${AlO}_{4}^{5-}$ anions dissolved in solution transform from four-coordinated aluminum ions to six-coordinated aluminum ions [Al(OH)_6_]^3−^ in alkaline environments, which react with Ca^2+^ and ${SO}_{4}^{2-}$ ions dissolved from FGDG to form ettringite [24].

Since the leaching characteristics of S/S materials under different environmental conditions are of high concern, numerous leaching test methods have been proposed. The system proposed by Lewin et al. [25] classifies leaching tests based on whether the leachant is renewed or not as either extraction tests such as TCLP, SPLP, HJ/T 299-2007 [26], and HJ 557-2010 [27] or dynamic tests such as ASTM C1308-08 [28]. While a single leaching test could not evaluate S/S efficiency scientifically, the leaching test series of the Leaching Environmental Assessment Framework (LEAF) developed by the United States Environmental Protection Agency (EPA, Washington, D.C., WA, USA) [29] were used to obtain more accurate estimates of the release of targeted contaminants from SSB S/S treated materials. Considering the low-permeability monolithic property of SSB S/S treated materials, the most appropriate methods are Method 1313, Method 1315, and Method 1316, which provide data to approximate local chemical equilibria as a function of the pH of the extract or the liquid–solid ratio, for evaluating the maximal leaching concentration, and the rate of release under conditions where mass transport dominates the rate of constituent release, respectively.

In the present study, tailings with high As concentrations were collected as the study objects, where the leaching risk of other hazardous elements was low after S/S treatment. The leaching tests performed on SSB S/S treated tailings (SST) include the Chinese extraction tests HJ/T299-2007 [26] and HJ557-2010 [27] and the LEAF series tests (Method1313, Method 1315, and Method 1316) [29] to investigate the effectiveness, leaching risk, and leaching mechanism of the SST. And the potential to reduce carbon emissions of utilization of SSB compared to cement was also discussed. The findings of this study could facilitate the utilization of steel slag in backfilling S/S processes, replacing cement to reduce CO_2_ emission.

## 2. Materials and Methods

### 2.1. Raw Materials

**Figure 1:**
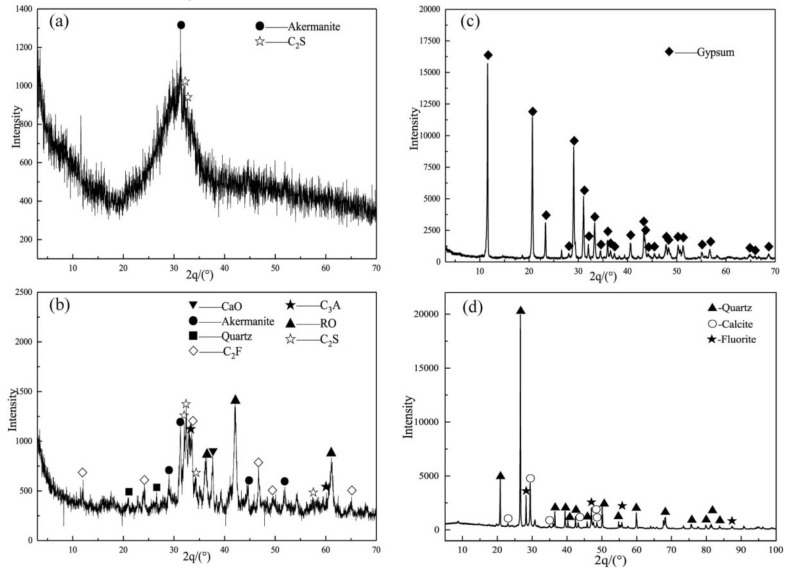
XRD patterns of the raw materials: (**a**) GGBFS; (**b**) SSP; (**c**) FGDG; (**d**) AT.

The SSBs were composed of SSP, GGBFS, and FGDG, pulverized and grounded to a specific surface area of 440, 435, and 350 m^2^/kg, respectively. The chemical components of the raw materials are listed in Table 1, and the XRD patterns are shown in Figure 1. The main phase of SSP included dicalcium silicate (C_2_S), tricalcium aluminate (C_3_A), dicalcium ferrite (C_2_F), akermanite, (MgO)_0.239_(FeO)_0.761_ (RO Phase), CaO, and quartz, which belonged to low-alkaline steel slag (alkalinity, M, of 2.05, Equation (1) [30]). The GGBFS consisted of abundant glassy phases and a small amount of akermanite and C_2_S, which was the superior product due to the activity coefficient K = 1.82 > 1.6 (Equation (2)) according to the Chinese standard GB/T203-2008 (Granulated Blast Furnace Slag Used for Cement Production). The main phase of FGDG was CaSO_4_·2H_2_O. A polycarboxylic acid water reducer (WR) was used to achieve an acceptable flowability of the mortar.

(1) $$M=W_{CaO}/\left(W_{SiO_{2}}+W_{P_{2}O_{5}}\right)$$

(2) $$K=\left(W_{CaO}+W_{MgO}+W_{Al_{2}O_{3}}\right)/\left(W_{SiO_{2}}+W_{MnO}+W_{TiO_{2}}\right)$$

Arsenic-containing tailings (AT) were collected from a closed lead and zinc tailings pond in Hechi Nandan, Guangxi (China). The main components of the tailings were quartz, calcite, and fluorite, of which the D_50_ and D_90_ particle sizes were 42 and188 μm, respectively. According to the XANES analysis results of tailings in the same tailings pond in the literature [31], the main forms of arsenic (As) in tailings are arsenopyrite (66 wt.%), arsenate (17 wt.%) and arsenite (17 wt.%). The total As concentration of the tailings was 2098 mg/kg as determined with an Agilent 7500a ICP-MS system (Agilent Technologies, Santa Clara, CA, USA) after aqua regia digestion (concentrated HNO_3_ and HCl in a ratio of 1:3 (*v*/*v*)) according to the Chinese standard method HJ 803-2016.

### 2.2. Preparation of SST

In the preparation of SSTs, the different constituents of SSBs were mixed with tailings at specific ratios as outlined in Table 2; although sufficient FGDG must be present for GGBFS hydration, excessive FGDG may lead to matrix expansion in the later stage of the hydration reaction; therefore, the proportion of FGDG was fixed at 10% [32]. These SSBs were mixed with the tailings and water according to the following procedure whilst maintaining the binders-to-tailings (B/T), water-to-solid (W/S), and WR-to-solid (WR/S) mass ratios of 0.25, 0.19, and 0.01, respectively, which also applied to the OPC S/S treated AT (PST) sample set as control group. SSB and tailings were mixed for 1 min using a standard cement mortar mixer; then, distilled water containing the dissolved WR was added, and the blended mortar was mixed for another 5 min before being cast into steel molds (40 mm × 40 mm × 160 mm). All samples were cured in a moist cabinet at (40 ± 2) °C and (90 ± 1)% relative humidity (similar to the underground filling environment in Guangxi [33]) for 3 d and then de-molded and placed under the same curing conditions until the appropriate degree of ageing was achieved.

The fluidity of the prepared SST samples and the PST sample was 300 mm, meeting the needs of artesian backfill, which was determined according to the Chinese standard GB/T 2419-2005 (Method for Determination of Fluidity of Cement Mortar). The unconfined compressive strength (UCS) of the samples was obtained according to GB/T 17671-1999 (Test Method for Strength of Cement Mortar (ISO method)). With the increase of the SSP content in the SSBs, the 3-d, 7-d, and 28-d UCS of the SST samples decreased while the 90-d UCS of the SST samples displayed a fluctuating behavior (Table 2). Based on the intention to use steel slag in large quantities and meeting the UCS requirement of underground backfilling (1-5 MPa for 28 d [4]), T-3 was chosen to investigate the characteristic As-leaching behavior based on the leaching tests.

### 2.3. Leaching Tests

#### 2.3.1. Extraction Tests

The single-batch extraction tests included a horizontal vibration method (HV) (HJ 557-2010, China). After being crushed and sieved to a particle size of less than 3 mm, the SST and PST samples were placed in deionized water with a liquid/solid ratio of 10 L/kg and were horizontally vibrated for 8 h at room temperature with an oscillation frequency of approximately 110 ± 10 per min before standing for a further 16 h. Furthermore, the sulfuric acid and nitric acid method (SN) (HJ/T299-2007, China) was employed. After being crushed and sieved to a particle size smaller than 9.5 mm, the SST and PST samples were placed in an extraction fluid with a liquid/solid ratio of 10 L/kg, prepared by adding a 2:1 (by mass) mixture of H_2_SO_4_ (18.4 N) and HNO_3_ (15.6 N) to deionized water until a target pH of 3.20 ± 0.05 was obtained; the sample mixtures were tumbled at (30 ± 2) rpm for 18 ± 2 h. These methods were employed on the SST and PST samples at a 3-d, 7-d, 28-d, and 90-d curing period. The As curing rate was calculated according to Equation (3):

(3) $$\mathrm{Curing}\;\mathrm{Rate}\;=\;\left(1\;-\;C_{S/S}/C_{T}\right)\;\times \;100\%$$

where *C_s/s_* is the concentration of As leached from SST and PST, μg/L; *C_T_* is the concentration of As leached from untreated tailings, μg/L.

#### 2.3.2. Leaching Environmental Assessment Framework Tests

##### Leaching Tests as per Method 1313

The leaching test was conducted as per Method 1313 of the EPA (“Liquid–Solid Partitioning (LSP) as a Function of Extract pH for Constituents in Solid Materials Using a Parallel Batch Extraction Procedure (LSP-pH)”) to investigate the influence of equilibrium pH of the leachate on characteristic leaching behavior. T-3 and PST samples were crushed and sieved to a particle size of less than 0.3 mm. Dilute nitric acid (2.0 N) and aqueous potassium hydroxide (1.0 N) solutions were used to adjust the pH, and samples were tumbled at (30 ± 2) rpm for (24 ± 2) h to achieve liquid–solid equilibrium. A liquid-to-solid ratio of 10 mL/g-dry was used for all batch tests. The final pH targets of the extracts were 2 ± 0.5, 4 ± 0.5, 5.5 ± 0.5, 7 ± 0.5, 8 ± 0.5, 9 ± 0.5, 10.5 ± 0.5, 12 ± 0.5, and 13 ± 0.5. Since the pH-dependent leaching of many elements may be sensitive to minor fluctuations in leachate pH, it is necessary to interpolate the test results of Method 1313 to the specified pH-target values to provide reproducible and comparative results. Interpolated leachate concentrations are obtained by standard linear interpolation of log-transformed data from two neighboring Method 1313 leachates (Equation (4)):

(4) $$\log C=\log C_{a}+\left(pH-pH_{a}\right)\;\times \;\left(\log C_{b}-\log C_{a}\right)/\left(pH_{b}-pH_{a}\right)$$

where log *C* is the log-transform of the leachate concentration interpolated to the target-pH value of 2, 4, 5.5, 7, 8, 9, 10.5, 12, or 13, log(mg/L); *pH_a_* and *pH_b_* are the measured pH value of leachate *a* and leachate *b* close to the target *pH* value; log *C_a_* and log *C_b_* are the log-transforms of the measured leachate concentration at *pH_a_* and *pH_b_*, respectively, log(mg/L).

##### Leaching Tests as per Method 1316

The leaching test was conducted as per Method 1316 of the EPA (“Liquid–Solid Partitioning (LSP) as a Function of Liquid–Solid Ratio for Constituents in Solid Materials Using a Parallel Batch Extraction Procedure (LSP-L/S)”) to investigate the influence of the liquid–solid ratio (L/S) on characteristic leaching behavior. T-3 samples were crushed and sieved to a particle size of less than 0.3 mm for 85 wt.% of the sample. The L/S was 10, 5, 2, 1, and 0.5 mL/g-dry with deionized water as leachant, and samples were tumbled at (30 ± 2) rpm for (24 ± 2) h.

##### Leaching Tests as per Method 1315

The leaching test was conducted as per Method 1315 of the EPA (“Mass Transfer Rates of Constituents in Monolithic or Compacted Granular Materials Using a Semi-dynamic Tank Leaching Procedure”) to investigate characteristic mass-transfer-based leaching behavior from a monolithic sample under completely saturated conditions whereby the leachant was renewed at the prescribed intervals. The T-3 and PST amples cured for 90 d (50 × 50 × 50 mm^3^) were completely immersed in deionized water in sealed vessels. The ratio of liquid to exposed surface area was maintained at 10 mL/cm^2^ for all samples. Leachates were collected at intervals of 0.8, 1, 2, 7, 14, 28, 42, 49, 63, and 112 d.

The flux (F) across the exposed surface and the observed effective diffusion coefficient (D_obs_) of the T-3 sample cured for 90 d (T-3-90d) and the PST sample cured for the same time (PST-90d) can be calculated by Equations (5) and (6); if diffusion is the dominant mechanism, de Groot and van der Sloot [34] suggested the relationship described by Equation (7).

(5) $$F_{i}=M_{i}/\left(t_{i}-t_{i-1}\right)$$

(6) Dobs=anAn/Δtn2×V/S2×Tn

(7) $$\log\left(B_{t}\right)\;=\;\frac{1}{2}\log\left(t\right)+\log\left(U_{max}d\sqrt{D_{obs}/\pi }\right)$$

where *F_i_* is the flux in interval i, mg/m^2^·s; *M_i_* is the mass released during the current leaching interval *i*, mg/m^2^; *t_i_* is the cumulative time at the end of the current leaching interval *i*, s; *t_i-1_* is the cumulative time at the end of the previous leaching interval *i-1*, s; *D_obs_* is the observed effective diffusion coefficient in chemically reactive systems, m^2^/s; *a_n_* is the mass released during leaching interval n, mg; *A_n_* is the initial concentration, mg; Δ*t_n_* is the leaching interval, days; *V* is the volume of the solidified monolithic samples, m^3^; *S* is the surface area of the solidified monolithic samples, m^2^; *T_n_* is the generalized mean of leaching time, s; *B_t_* is the cumulative maximum release of the compound, mg/m^2^; *U_max_* is the maximum leachable quantity, mg/kg; *d* is the bulk density of the S/S product, kg/m^3^.

After conducting leaching test Method 1315, the UCS of the monolithic samples was tested to investigate their physical stability. All leachates were filtered through 0.45-μm polypropylene membrane filters, preserved with HNO_3_, and stored at 4 °C. As concentrations were determined by a PerkinElmer Optima 8300 ICP-OES (Perkin Elmer, Cumberland, NJ, USA) or an Agilent 7500a ICP-MS (Agilent Technologies, Santa Clara, CA, USA) system. Method blanks (deionized water), analytical blanks, and replicates were prepared and analyzed for quality control purposes.

## 3. Results

### 3.1. Extraction Tests Results

**Figure 2:**
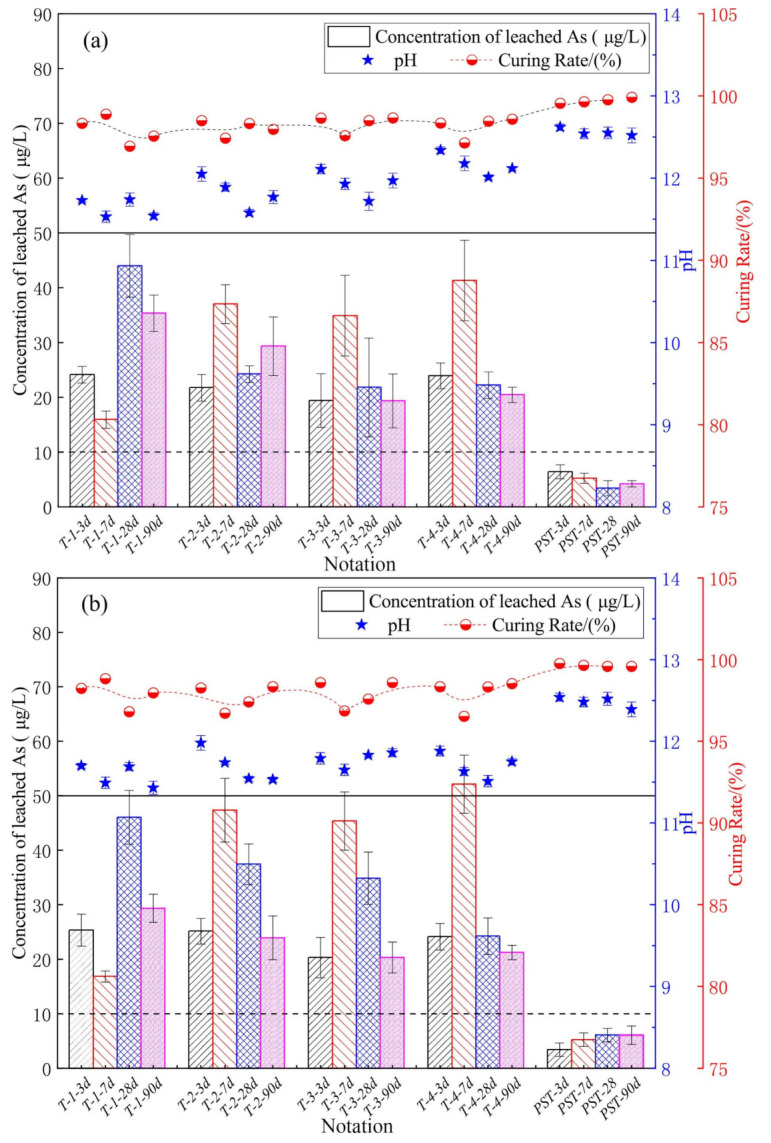
Concentration of leached As, leachate pH, and As curing rate of (**a**) the horizontal vibration method tests; (**b**) the sulfuric acid and nitric acid method tests.

Figure 2 shows the results of the single-batch extraction tests. All leached-As concentrations of SST samples were below the type IV water threshold of the Chinese standard as published in the Standard for Groundwater Quality (GB/T 14848-2017) (50 μg/L, transverse solid line) with the exception of T-4-7d (52 μg/L), while the leached-As concentrations of the PST samples were below the type III water threshold (10 μg/L, transverse dotted line). The difference of the leached-As concentrations of samples cured for 3, 7, 28, and 90 d indicated that the hydration reaction affected the As S/S. The leachate pH values for SST and PST samples were in the range of 11.43–12.12 and 12.39–12.62, respectively. With the increase in SSP content in SSB, the leachate pH increased due to the intrinsically high pH of SSP. The As curing rate (Equation (3)) for SST and PST samples were in the range of 96.80–98.89% and 99.52–99.92%, respectively.

### 3.2. Results of pH-Dependent LSP

**Figure 3:**
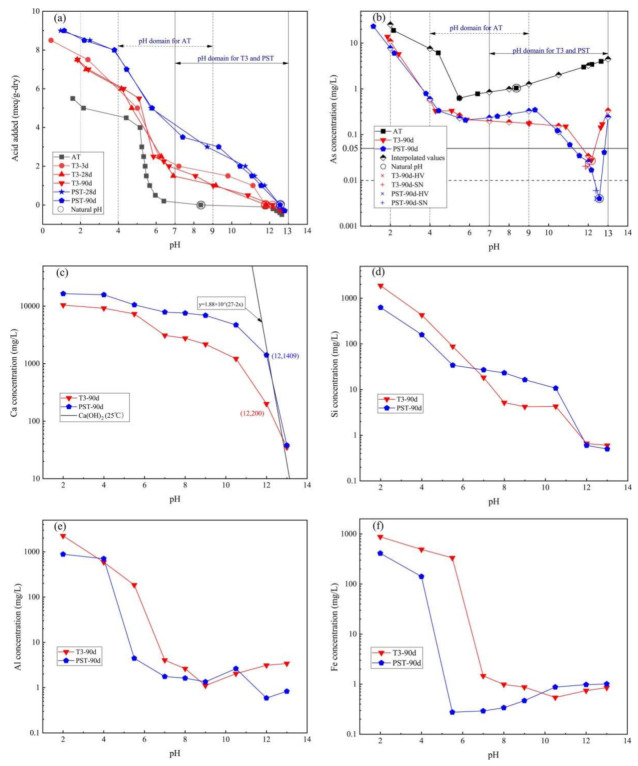
Method 1313 results for (**a**) acid/base neutralization capacity; (**b**) As concentrations; (**c**) Ca concentrations; (**d**) Fe concentrations; (**e**) Al concentrations; (**f**) Si concentrations as a function of leachate pH for T-3-90d and PST-90d.

Figure 3 presents the acid/basic neutralization capacity (ANC/BNC) and LSP As, Ca, Si, Al, and Fe concentrations as a function of the leachate pH value. The natural pH of T-3 (3 d, 11.79; 28 d, 11.83; 90 d, 12.16) and P (28 d, 12.55; 90 d, 12.56) increased sharply compared to the untreated AT (8.36), and the ANC (leachate pH = 7) of T-3 (~2 meq/g-dry) and P (~4 meq/g-dry) significantly improved in comparison to the untreated AT (~0 meq/g-dry). Accounting for the complete exposure of the tailings into acid rain and acid mine wastewater caused by pyrite oxidation, the applicable pH domain of 4–9 for AT was chosen rather than the default 5.5–9 used for the natural pH of soil, while the pH domain for T-3-90d and PST-90d was chosen as 7–13 to capture the natural pH and anticipated environmental processes (e.g., carbonation) that may occur over time (Figure 3a,b).

Figure 3b illustrates the pH-dependent As equilibrium concentration of AT, T3-90d, and PST-90d. The As concentration of AT increased continuously from 1.044 mg/L (natural pH 8.36) with added KOH and reached 4.476 mg/L at pH 13 due to the dissolution of arsenopyrite (Equation (8)) and the deprotonation in alkaline solution because of the amphoteric property of As [35]. With decreasing pH, the As concentration of AT decreased to the lowest value at pH 5.5 (0.626 mg/L) and then increased to the highest value at pH 2 (25.491 mg/L), which was attributed to the adsorption of iron and aluminum oxides/hydroxides on As in different valence states and the dissolution of the oxides/hydroxides as the pH went down [36]. The As equilibrium concentration of T-3 and PST was between one and two orders of magnitude lower than that of AT over a pH range of 2–13 due to the reaction of hydration products of SSB and OPC with As in AT; the As concentration of T-3-90d was 0.0276 mg/L at natural pH 12.16, while that of PST-90d was 0.004 mg/L at natural pH 12.56. The HV and SN leaching test results are also presented, which data sets were close to the equilibrium curves, indicating that the extraction test results depended significantly on the pH of the leachate.

(8) $$2\mathrm{FeAsS}+10OH^{-}+7O_{2}=Fe_{2}O_{3}+2AsO_{4}^{3-}+2SO_{4}^{2-}+5H_{2}O$$

Figure 3c–f present the equilibrium concentrations of the main elements of T3-90d and PST-90d as a function of leachate pH by interpolation to the target pH for clarity (Equation (4)), which were closely related to the release of As. The Ca concentrations of natural T3-90d and PST-90d were 199 and 803 mg/L, respectively, which increased with decreasing pH and decreased when base was added. The line in Figure 3c represents the solubility equilibrium curve of Ca(OH)_2_ at 25 °C (Equation (9)), indicating that the solubility of Ca(OH)_2_ controlled the Ca release over pH 12–13 for PST-90d and at pH 13 for T3-90d, leading to the decline in Ca concentration to 38 mg/L and 35 mg/L, respectively, at pH 13. The natural pH for PST-90d was close to the pH of 12.60 for saturated Ca(OH)_2_ solution at 25 °C because the hydration products of OPC contain Ca(OH)_2_ (~25 wt.% [37]); since the hydration reaction of SSB only contributes to a very small amount of Ca(OH)_2_ [11], the leachate was unsaturated in Ca for T3-90d with added KOH over the pH range of 12–13. Figure 3d shows that the Si concentration increased with the addition of acid, except for the observed plateaus for T-3-90d and PST-90d over the pH ranges of 5.5–11 and 8–11, respectively.

(9) $$K_{sp}=C_{Ca^{2+}}\times C_{oH^{-}}^{2}=C_{Ca^{2+}}\times {\left(10^{pH-14}\right)}^{2}\to \;\;M_{Ca^{2+}}=1.88\times 10^{27-2pH}\;\left(25\;{}^{\circ}C\right)$$

Figure 3e shows the rapid increase in Al concentration of T3-90d and PST-90d below pH 4 in accordance with the aluminum hydroxide solubility equilibrium, which adsorbed As effectively at pH 4–7.7 [38,39], while Al concentrations did not increase sharply at pH > 9, where the C-A-S-H and ettringite influenced the release of Al [40]. The Fe concentration in solution as a function of pH is often a useful indicator of the redox state of a system. At pH values between 4.8 and 10, the Fe^3+^ concentration would remain below 10 μg/L due to the Fe(OH)_3_ solubility equilibrium while Fe(OH${)}_{4}^{-}$ appeared at pH > 10, increasing the Fe concentration. Thus, the Fe concentration presented in Figure 3f indicated the presence of moderately and weekly reducing conditions for T3-90d and PST-90d, respectively; according to the results of geochemical speciation modeling [41], these conditions were caused by the different states of Fe in SSP (Fe, FeO, Fe_3_O_4_, Fe_2_O_3_, 2CaO·Fe_2_O_3_, and MgO·2FeO [42]) and OPC (4CaO·Al_2_O_3_·Fe_2_O_3_).

### 3.3. Results of L/S-Dependent LSP

**Figure 4:**
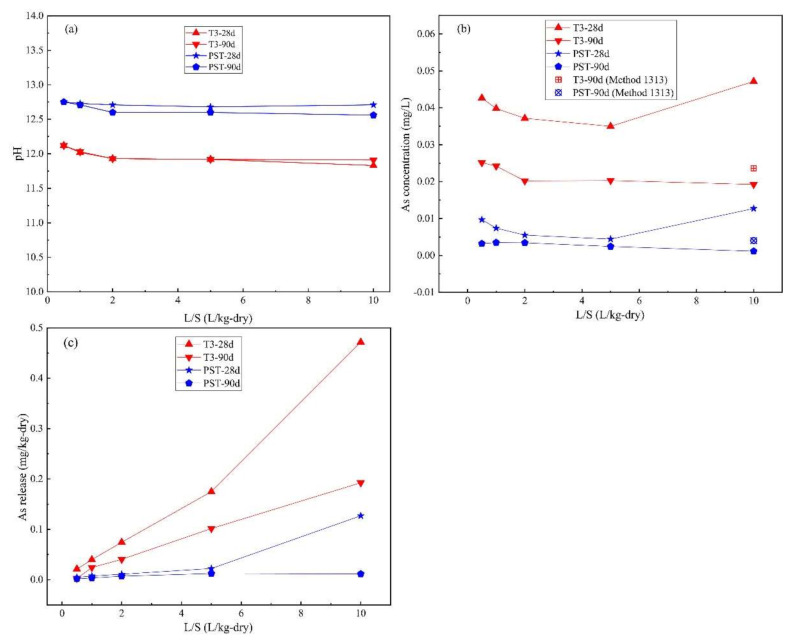
Method 1316 results for (**a**) pH; (**b**) As concentration; (**c**) As release from T3 and PST at curing for 28 d and 90 d.

Figure 4 presents the As LSP behavior as a function of L/S, where the leachate pH of T-3 and PST remained at ~12 and ~12.6 respectively, over the L/S range of 0.5–10. The As concentration of T3-90d and PST-90d decreased and then remained unchanged at L/S ≥ 2 as the L/S increased. The As concentration of T3-28d and PST-28d first decreased in a similar way compared to the samples cured for 90 d but exhibited an upward trend at L/S ≥ 5 with significantly higher values at an L/S of 10, which is consistent with the characteristic As leaching behavior in the pH range of 12–13 (Figure 3b).

### 3.4. Results of Mass Transfer Rates

**Figure 5:**
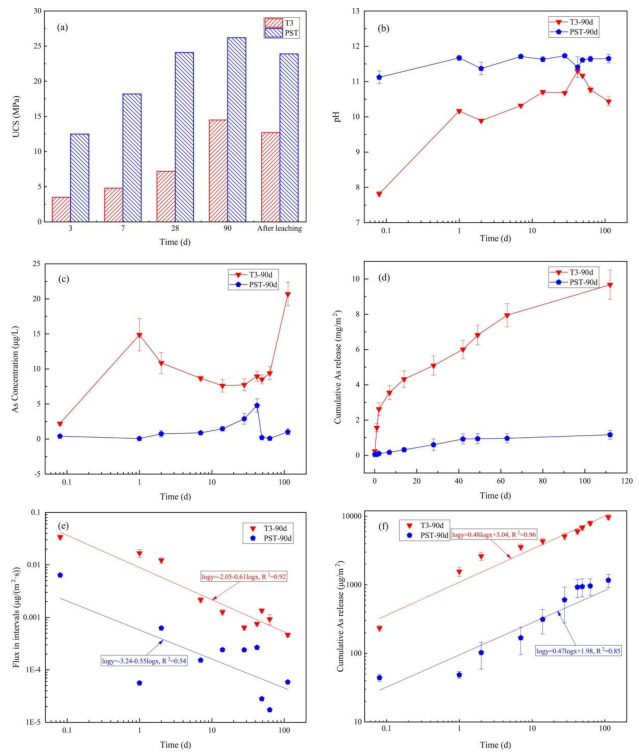
Method 1315 results: (**a**) comparison of UCS values of samples with different curing times to the results after 112 d of semi-dynamic leaching; (**b**) pH; (**c**) As concentration; (**d**) cumulative As release; (**e**) flux in the different intervals; (**f**) log-log plot of cumulative As release over time for T3-90d and PST-90d.

Figure 5a shows the UCS of T3 and PST at curing times of 3, 7, 28, and 90 d and after 112 d of semi-dynamic leaching tests. The UCS of P reached its highest value after 28 d of curing, while the UCS of T3 still greatly improved from 28 d to 90 d. After the semi-dynamic leaching tests, the UCS of T3-90d and PST-90d decreased by 12.14% and 8.78%, respectively. Figure 5b,c show the leachate pH and As concentrations of every interval, where the pH of PST-90d fluctuated at 11.5 and that of T3-90d ranged from ~10 to ~11 except at the first 2-h leaching interval (pH 7.81), and the As concentrations of T3-90d and PST-90d ranged from 2–20 and 0–5 μg/L, respectively, which were far lower than the pH-dependent LSP results (Figure 3b), indicating that the “dilute” boundary condition was met for As mass transfer. The growth rate of cumulative As release of T3-90d and PST-90d decreased gradually with extended leaching time (Figure 5d), and the cumulative As release of T3-90d reached 9.674 mg/m^2^ at 112 d, which was 8.28 times higher than that of PST-90d (1.168 mg/m^2^, Table 3). Log-transformed As flux (Equation (5)) during the intervals and log-transformed cumulative As release are presented in Figure 5e,f, respectively, as a function of the log-transformed leaching time. The log-log plot of cumulative As release and leaching time of T3-90d and PST-90d can be fitted by a straight line throughout the entire leaching duration with slopes of 0.48 (R^2^ = 0.96) and 0.47 (R^2^ = 0.85), respectively, indicating that the As leaching mechanisms were similarly controlled by diffusion.

## 4. Discussion

This study demonstrates that the usage of SSP as the main component of binders for AT S/S treatment is feasible based on the results of UCS, the extraction tests, and the LEAF series tests. Only single-point leaching data could be provided by the extraction leaching tests, and the results are greatly controlled by the pH of the leachate. Notably, LEAF leaching tests could provide more scientific information. Combining Method 1313 and Method 1316 test results, full LSP screening assessment demonstrated that the LSP limits of AT, T3-90d, and PST-90d were controlled by solubility according to Equation (10) [43], and the highest concentrations over the pH and L/S range were 7.56 (at pH 4), 0.34 (at pH 13), and 0.33 (at pH 9) mg/L, respectively. When considering the effect of a relatively small volume of leachate interacting with a larger groundwater body and associated transport toward a down-gradient exposure point through the use of dilution and attenuation factors (DAF), a DAF value of 10 was assumed for oxyanions when risk assessment values were not available [44]. The DAF-related leaching assessment ratio (AR_DAF_) of AT, T3-90d, and PST-90d were 75.6, 3.4, and 3.3, respectively (Table 4), indicating that AT poses a high environmental pollution risk and SSB with 80 wt.% SSP is highly effective on S/S of AT and equally effective compared to OPC. However, the above results were based on the LSP-pH and LSP-L/S equilibrium analysis, revealing the maximum degree of leaching. The monolithic property of S/S waste controlling the leaching rate was not considered. Method 1315 leaching test results showed that As leaching from monolithic T3-90d and PST-90d was controlled by diffusion with very low values of D_obs_ (T3-90d, 9.35 × 10^−15^ cm^2^/s; PST-90d, 1.55 × 10^−16^ cm^2^/s), which could be applied for “controlled utilization” [45].

(10) $$If\;\frac{C_{\max\left(pH2,\;9,\;13\right)}\times \left(1-0.28\right)}{C_{\max\left(pH\;domain\right)}\times \left(1+0.28\right)}>1,\;LSP\;limit\;was\;controlled\;by\;solubility$$

The results of the Method 1313 leaching tests also included Ca, Si, Al, and Fe leaching data related to the As release at final leachate pH ranges of 2–13 for T3-90d and PST-90d (Table 5), which provided an approach to investigate the S/S mechanism. As reported in literature, Ca–As co-precipitation considerably influenced the release of As at alkaline pH in S/S matrices (Equations (11)–(13)) [33], and As was dissolved in large quantities at Ca concentrations below 100 mg/L [46]. Thus, the characteristic As leaching properties of T-3-90d and PST-90d over pH 12–13 (Figure 3b) was controlled by the equilibrium between Ca–As co-precipitation and dissolution and influenced by the Ca equilibrium concentrations of Ca(OH)_2_. The hydration products of SSB and OPC, ettringite and C–A–S–H, were dissolved in the pH range of 7–12 (Table 5) [47], while As leaching did not increase with the leaching of Ca, Si, and Al at pH 7–10.5, possibly because the products related to the hydration of As first dissolved and were then fixed by Fe(OH)_3_ gels. The moderately reducing property of SSP would reduce As(V) to As(III), but the As concentrations of T-3-90d in the pH range of 2–10.5 did not increase compared with PST-90d (Figure 2b), indicating that Fe(III) would stabilize As(V) and As(III) at pH > 4. Other techniques, such as microanalysis, are needed to verify the controlling S/S mechanism at every final leachate pH interval to identify a method to promote S/S efficiency.

(11) $$5{\mathrm{Ca}}^{2+}+3{\mathrm{AsO}}_{4}^{3-}+{\mathrm{OH}}^{-}={\mathrm{Ca}}_{5}{\left({\mathrm{AsO}}_{4}\right)}_{3}\left(\mathrm{OH}\right),{\;k}_{sp}=10^{-40.12}\;(25\;{}^{\circ}C)$$

(12) $$4{\mathrm{Ca}}^{2+}+2{\mathrm{AsO}}_{4}^{3-}+2{\mathrm{OH}}^{-}={\mathrm{Ca}}_{4}{\left(\mathrm{OH}\right)}_{2}{\left({\mathrm{AsO}}_{4}\right)}_{2}\cdot 4H_{2}O,k_{sp}\;=10^{-27.49}\;\left(25\;{}^{\circ}C\right)$$

(13) $${\mathrm{Ca}}^{2+}\;+{\;\mathrm{HAsO}}_{3}^{2-}\;={\;\mathrm{CaHAsO}}_{3},{\;k}_{sp}\;=10^{-6.52}\;(25\;{}^{\circ}C)$$

The main greenhouse gas component of cement industry is CO_2_. According to different emission sources, it includes the process emissions generated by carbonate decomposition and the small amount of organic carbon combustion in the raw material, fuel combustion emissions, and indirect emissions generated by power consumption in the production process [48]. The cement carbon emission factor (CO_2_ emission per unit of cement produced) ranges from 0.65 to 0.92 t CO_2_/t-cement [49,50,51]. The carbon emission of SSB mainly comes from the indirect emissions generated by power consumption in the grinding process. The CO_2_ emission factor of electricity consumption is about 1 kg CO_2_/KWh. According to the different grinding processes, the power consumption per unit of steel slag powder converted to 450 m^2^/kg is about 31-59 kWh/t [52]. Blast furnace slag and flue gas desulphurization gypsum is easier to grind than steel slag. Even if the CO_2_ emissions of SSB is calculated according to the grinding energy consumption of steel slag, it is about 0.031–0.059 t CO_2_/t-SSB, which is much lower than cement. Thus, the utilization of SSB in S/S process has great potential in carbon reduction.

## 5. Conclusions

In the present study, a series of leaching tests were performed on SST to evaluate the feasibility of SSB as binder for AT S/S. Compared with the single-batch extraction leaching tests, the multi-process LEAF tests could present more detailed and scientific information on the waste leaching risk and for the evaluation of the S/S effectiveness of the binders. Conducting leaching tests over a range of pH values provided an approach to investigate the S/S mechanism. Based on the results obtained from this study, the following conclusions can be drawn:

- The extraction test results showed that the As curing rate for SST and PST samples were in the range of 96.80–98.89% and 99.52–99.2%, respectively.
- The LSP limits of AT, T3-90d, and PST-90d were controlled by solubility, and the highest concentrations over the investigated pH and L/S range were 7.56 (at pH 4), 0.34 (at pH 13), and 0.33 (at pH 9) mg/L, respectively.
- The As leaching mechanism of monolithic SST (T3-90d) was controlled by diffusion, and the mean D_obs_ of T3-90d of 9.35 × 10^−15^ cm^2^/s was higher than that of PST-90d (1.55 × 10^−16^ cm^2^/s).
- As leaching of SST (T3-90d) and PST (PST-90d) was controlled by the equilibrium between Ca–As co-precipitation and dissolution and influenced by the Ca equilibrium concentrations of Ca(OH)_2_ when the leachate pH was in the range of 12–13. Furthermore, As leaching was strongly correlated to Fe-ion leaching when the leachate pH was less than 10.5.
- The utilization of SSB in S/S process has great potential in carbon reduction.

## Figures and Tables

**Table 1:** Chemical components (wt.%) and pH value of the raw materials.

Notation	SiO_2_	TiO_2_	Al_2_O_3_	Fe_2_O_3_	MnO	P_2_O_5_	MgO	CaO	Na_2_O	K_2_O	SO_3_	As_2_O_3_	F	LOI	pH
AT	53.23	0.12	5.07	1.65	0.7	0.1	1.17	25.49	-	1.38	1.12	0.18	6.05	10.61	7.64
SSP	17.05	0.91	5.73	22.33	3.63	1.72	9.01	38.42	0.16	0.09	-	-	-	1.42	11.96
GGBFS	33.32	0.85	15.43	1.01	0.52	0.05	10.78	36.89	0.49	0.38	-	-	-	0.12	11.78
FGDG	2.03	0.04	0.78	0.48	0.03	0.06	1.04	30.01	0.06	0.15	44.97	-	-	22.07	7.68

**Table 2:** Formulations for SSB, SST, and PST.

Notation	Steel Slag-Based Binder (Mass Fraction/ wt.%)			B/T (*w*/*w*)	W/S (*w*/*w*)	WR/S (*w*/*w*)	Fluidity (mm)	UCS (MPa)			
	SSP	GGBFS	FGDG					3 d	7 d	28 d	90 d
T-1	60	30	10	0.25	0.19	0.01	300	9.1	12.2	14.3	15.1
T-2	70	20	10				300	6.3	8.1	10.1	13.2
T-3	80	10	10				300	3.5	4.8	7.2	14.5
T-4	90	0	10				300	1.1	1.9	2.5	9.8
PST ^1^	100						300	12.5	18.2	24.1	26.2

^1^ OPC as binder for control group.

**Table 3:** Cumulative As release, mean Dobs, and log-transformed fitting curve equation.

Sample	Cumulative Release /(mg/m^2^)			Fitting Curve Equation	R^2^	Slope	Mechanism	Mean Dobs /(cm^2^/s)
	28 d	63 d	112 d					
T3-90d	5.091	7.943	9.674	logy = 0.48logx + 3.04	0.96	0.48	diffusion	9.35 × 10^−15^
PST-90d	0.603	0.964	1.168	logy = 0.47logx + 1.98	0.85	0.47	diffusion	1.55 × 10^−16^

**Table 4:** Full As LSP screening assessment, leaching assessment ratios (ARs), and leaching ARs considering dilution and attenuation (ARDAF) for AT, T3-90d, and PST-90d.

As	Threshold Value ^1^ /(mg/L)	Total Content /(mg/kg-dry)	Method 1313					Method 1316		AR ^2^	AR_DAF_^3^
			Available Content /(mg/L)	pH for Available Content	Max Conc. Over pH Domain /(mg/L)	pH at Max Conc.	Limitation by Available Content or Solubility	Max. Conc. over L/S Range	L/S at Max. Conc.		
AT	0.01	2098	25.49	2	7.56	4	Solubility	-	-	756	75.6
T3-90d	0.01	1376	10.96	2	0.34	13	Solubility	0.025	0.5	34	3.4
PST-90d	0.01	1376	7.78	2	0.33	9	Solubility	0.004	1	33	3.3

^1^ The type III water threshold of the Chinese standard published in the Standard for Groundwater Quality (GB/T 14848-2017); ^2^ AR = C_max_/Threshold value; ^3^ AR_DAF_ = AR/DAF.

**Table 5:** Mean concentrations of leached As, Ca, Si, Al, and Fe and growth trend at continuous pH intervals for T3-90d and PST-90d after Method 1313 leaching tests.

	pH	13–12		12–10.5		10.5–9		9–7		7–5.5		5.5–4		4–2	
	Element	MLC ^1^	Trend	MLC ^1^	Trend	MLC ^1^	Trend	MLC ^1^	Trend	MLC ^1^	Trend	MLC ^1^	Trend	MLC ^1^	Trend
T3-90d	As	0.203	↘	0.114	↗	0.169	→	0.187	→	0.224	→	0.373	↗	5.761	↗
	Ca	117	↗	709	↗	1695	↗	2679	↗	5211	↗	8250	↗	9799	→
	Si	0.63	→	2.48	↗	4.258	↗	9.259	↗	53.12	↗	257.8	↗	1155	↗
	Al	3.261	→	2.575	↘	1.573	↘	2.588	↗	94.56	↗	386.7	↗	1420	↗
	Fe	0.795	→	0.642	→	0.701	→	1.106	→	162.5	↗	411.7	↗	684.1	↗
PST-90d	As	0.066	↘↗	0.059	↗	0.228	→	0.274	→	0.224	→	0.388	↗	3.800	↗
	Ca	723.5	↗	3052	↗	5794	↗	7435	→	9201	↗	13,152	↗	16,120	→
	Si	0.55	→	5.66	↗	13.56	↗	22.21	→	30.55	→	96.28	↗	392.7	↗
	Al	0.709	→	1.610	↗	1.989	↘	1.572	→	3.098	↗	353.1	↗	791.3	→
	Fe	0.995	→	0.929	→	0.665	→	0.356	→	0.286	→	70.65	↗	275.5	↗

^1^ MLC is the mean leaching concentration, mg/L.

## Data Availability

Not applicable.
